# Topical estrogen application promotes cutaneous wound healing in db/db female mice with type 2 diabetes

**DOI:** 10.1371/journal.pone.0264572

**Published:** 2022-03-10

**Authors:** Kanae Mukai, Shin-ichi Horike, Makiko Meguro-Horike, Yukari Nakajima, Arya Iswara, Toshio Nakatani

**Affiliations:** 1 Faculty of Health Sciences, Institute of Medical, Pharmaceutical and Health Sciences, Kanazawa University, Ishikawa, Japan; 2 Research Center for Experimental Modeling of Human Disease, Kanazawa University, Ishikawa, Japan; 3 Division of Health Sciences, Department of Clinical Nursing, Graduate Course of Nursing Science, Graduate School of Medical Sciences, Kanazawa University, Ishikawa, Japan; Indiana University Purdue University at Indianapolis, UNITED STATES

## Abstract

Female sex hormones are beneficial effects for wound healing. However, till date, whether topical estrogen application can promote cutaneous wound healing in diabetes remains unclear. Therefore, the present study aimed to validate the effect of topical estrogen application on cutaneous wound healing in a type 2 diabetes db/db mice model. In total, 22 db/db female mice with type 2 diabetes and eight C57BL/6J female mice were subjected to two full-thickness wound injuries. The mice were divided into the db/db, db/db + estrogen, db/db + vehicle, and wild type (WT) groups. Wound healing was assessed until day 14. The db/db group had a significantly high wound area ratio (wound area/initial wound area) on days 3–14 and a significantly low re-epithelialization ratio on days 7 and 14. Moreover, their angiogenesis ratio was significantly low on day 7 and high on day 14. In contrast, compared with the db/db group, the db/db + estrogen group had a significantly lower wound area ratio on days 1–14 and angiogenesis ratio on day 14, thereby indicating early withdrawal of new blood vessels, as well as a significantly higher re-epithelialization ratio on days 7 and 14 and Ym1^+^ M2 macrophage/macrophage ratio on day 7. Moreover, microarray analysis showed that the top 10 upregulated or downregulated genes in the db/db group were reversed by estrogen treatment, particularly on day 14, in comparison with the WT group. Thus, topical estrogen application reduced the wound area, promoted re-epithelialization and angiogenesis, and increased the number of M2 macrophages in mice with type 2 diabetes. Furthermore, it improved the differential regulation of genes in db/db mice. Therefore, such treatment can enhance cutaneous wound healing in female mice with type 2 diabetes.

## Introduction

Chronic nonhealing wounds are a major health and economic burden worldwide. In developed countries, their prevalence rate is 1%–2% in the general population [1, 2], and they affect the quality of life in nearly 2.5% of the total population in the United States [3]. Based on an economic survey on chronic nonhealing wounds, the expenditures associated with wound care are substantially higher than those previously recognized [4], and the global advanced wound care market is projected to reach $18.7 billion by 2027 [5]. Among the different types of chronic nonhealing wounds, diabetic foot ulcer (DFU) is associated with high morbidity [6] and is extremely difficult to treat. Moreover, DFU is associated with a high amputation and mortality rate [7, 8].

In the 1990s, female sex hormones were found to have beneficial effects on wound healing. Wounds heal poorly in postmenopausal women with systemically low estrogen levels. Meanwhile, hormone replacement therapy can reverse delayed wound healing [9], and age-associated delayed wound healing can be managed by topical estrogen replacement in healthy aging humans [10]. Moreover, genetically, microarray analysis revealed that estrogenic sex hormones play an important role in cutaneous wound healing [11]. In animal studies, the administration of estrogen can promote wound healing based on a molecular biology perspective [12–16].

Male sex was associated with nonhealing of DFU after lower extremity amputation [17]. In animal studies, estrogen treatment was found to promote wound healing in mice with diabetes [18, 19]. By contrast, another study reported that only estrogen treatment did not promote wound healing in mice with diabetes [20]. The selective blockade of estrogen receptor beta can improve wound healing in mice with diabetes [21]. However, till date, whether estrogen application promotes cutaneous wound healing in diabetes remains unclear.

Diabetes mellitus is a chronic disease characterized by a relative or absolute lack of insulin, resulting in hyperglycemia. Moreover, it is classified into two types. Type 1 diabetes is caused by the autoimmune destruction of pancreatic beta cells, which produce insulin. Type 2 diabetes is attributed to insulin resistance along with the failure of beta cells to compensate. Various animal diabetes models that have been established include the following: chemically induced, spontaneous autoimmune or genetically induced models of type 1 diabetes and genetically induced or high-fat feeding models of type 2 diabetes [22, 23]. Among these, genetically induced db/db or ob/ob mice are widely used in animal models in the field of cutaneous wound healing in diabetes [24]. Michaels et al. revealed that db/db mice exhibit severe wound healing impairment compared with other murine strains with diabetes. Therefore, they are an appropriate model for assessing interventions for diabetic wound healing [25]. The present study aimed to validate the effect of topical estrogen application on cutaneous wound healing in a type 2 diabetes db/db mouse model.

## Materials and methods

### Animals

In total, 22 10-week-old female db/db mice (C57BLKS/J Iar-+Lepr^db^/+Lepr^db^) with diabetes and 8 10–12-week-old female C57BL/6J mice (Sankyo Lab Service Co., Tokyo, Japan) were included in the experiments. They were individually placed in a cage in an air-conditioned room at 25.0 ± 2.0°C with lights turned on from 08:45 to 20:45, and water and chow were provided freely. All animal experiments conducted in this study were reviewed and approved by the Kanazawa University Animal Experiment Committee. Moreover, they were performed in accordance with the Guidelines for the Care and Use of Laboratory Animals of Kanazawa University, Japan (AP-163749).

### Wounding and blood glucose assay

The mice were anesthetized using 1.5% isoflurane (Wako Pure Chemical Industries Ltd., Tokyo, Japan) at 1.5 L O_2_/min via a plastic tube mask. The dorsum was shaved, and the mice were divided into the diabetic (db/db), topical estrogen wound treatment diabetic (db/db + estrogen), vehicle wound treatment diabetic (db/db + vehicle), and wild-type (WT) groups. In the present study, WT C57BL/6J mice were used as the comparator group to determine the effect of estrogen application on cutaneous wound healing in db/db mice compared with WT mice with normal wounds. Before shaving, an aliquot of blood was obtained from the tail vein, and the blood glucose level (mg/dL) of mice in each group was monitored using an automatic glucometer (Carefast^®^, NIPRO, Tokyo, Japan) before and 2–12 h after fasting. One day after shaving, two circular full-thickness skin wounds (with a diameter of 4 mm) in the panniculus carnosus muscle were created on both sides of the dorsum with a Kai sterile disposable biopsy punch (Kai Industries Co. Ltd., Gifu, Japan) in the mouse under anesthesia. The wounds were covered with a hydrocolloid dressing (Tegaderm; 3M Health Care, Tokyo, Japan) to maintain a moist environment, and the mouse was then wrapped with sticky bandages (3M^™^ Kind Removal Silicone Tape; 3M Health Care), which were changed daily. The blood glucose level and the body weight of each group were monitored daily until day 14 after wounding.

### Exogenous estrogen administration

Estradiol benzoate (estra-1,3,5(10)-triene-3,17β-diol 3-benzoate) (OVAHORMON^®^INJECTION; ASKA Pharmaceutical Co. Ltd., Tokyo, Japan) at a dose of 0.75 μg/g/day was applied to the wounds daily according to our previous study [26]. Estradiol benzoate was diluted at 0.75 μg/g in sesame oil (Wako). The dose of sesame oil, which was used as a vehicle, was similar to that of estradiol benzoate, and it was applied on the wound daily.

### Macroscopic observations

Day 0 was defined as the day when wounds were established, and the process of wound healing was assessed until day 14 after wounding. Wound edges were traced using polypropylene sheets, and images were obtained every day with the animals under inhalational anesthesia. The tracing on the sheets was captured using a scanner onto a personal computer using Adobe Photoshop Elements 11.0 (Adobe System Inc., Tokyo, Japan), and the wound areas were assessed using ImageJ (National Institutes of Health, Bethesda, MD, USA). Based on our previous studies, the wound area was presented as the ratio of daily wound area to the initial wound area on day 0 [16, 26, 27].

### Uterus assay

The mice were euthanized by overdosing with isoflurane on day 14 after wounding. The uterus was harvested according to the Organisation for Economic Co-operation and Development guidelines, and its wet weight was evaluated.

### Tissue collection

The mice were euthanized by overdosing with isoflurane on days 7 and 14 after wounding. The wound and surrounding intact skin were harvested, and each wound and surrounding intact skin samples were bisected. One-half of each wound was embedded in tissue-Tek OCT (Sakura Finetek Japan Co., Ltd., Tokyo, Japan) before fixing for histology. The remaining half was snap-frozen in liquid nitrogen and was stored at −80°C before RNA isolation.

### Immunohistological and immunofluorescence staining

At least three serial ice sections near the center of the wound were obtained from a wound and stained. Next, 5-μm-thick ice sections were subjected to hematoxylin and eosin (H&E) staining, and they were immunohistologically stained with an anti-CD31antibody (550274, BD Pharmingen, Tokyo, Japan) to detect blood vessels, an anti-Ym1 antibody (#01404, StemCell Technologies, Tokyo, Japan) to detect M2 macrophages, and an anti-Mac-3 antibody (550292, BD Pharmingen) to detect macrophages. Negative control slides were obtained by omitting each primary antibody.

For anti-CD31 antibody immunohistological staining, sections were fixed for 15 min in ice-cold acetone, washed with tris-buffered saline, and incubated with 5% BSA and 5% skim milk. Rat anti-CD31, which is the primary antibody, was used at a dilution of 1:50 and was incubated for 1 h. Subsequently, the specimens were incubated with secondary antibody goal polyclonal to Rat IgG H&L (AP) (ab6846; Abcam, Cambridge, UK) at a dilution of 1:500 at room temperature for 2 h. Then, they were incubated using the BCIP/NBT substrate system (Agilent Technologies, Santa Clara, CA, USA) for 10 min until staining was achieved.

In anti-Ym1 and anti-Mac-3 antibody immunofluorescence staining, sections were fixed overnight in 4% paraformaldehyde, washed with phosphate-buffered saline, and incubated with a protein blocking solution (X0909; Agilent). The rabbit anti-Ym1 and the rat anti-Mac-3, which are the primary antibodies, were used at a dilution of 1:100 and incubated overnight. Subsequently, the specimens were incubated with Alexa Fluor 647-conjugated goat anti-rabbit IgG H&L (ab150079; Abcam) and Alexa Fluor 488-conjugated goat anti-Rat IgG H&L (ab150157; Abcam), which are secondary antibodies, at a dilution of 1:500 at room temperature for 2 h and counterstained with 4’, 6-diamidino-2-phenylindole (DAPI).

### Microscopic observations

H&E staining and anti-CD31 antibody immunohistological staining images were imported onto a computer using a digital microscopic camera (DP2-BSW; Olympus, Tokyo, Japan). The re-epithelialization ratio (re-epithelialization length/wound length) was assessed using DP2-BSW Olympus. The ratio of anti-CD31 antibody-positive blood vessels (anti-CD31-positive blood vessel pixels/total wound pixels) was evaluated using Adobe Photoshop Elements 11.0. The region of interest of the wound extended from the wound edges below the necrotic tissues or the new epithelium to the level of the panniculus carnosus. Anti-Ym1 and anti-Mac-3 antibodies immunofluorescence staining images were imported onto a computer using a digital microscopic camera (FLUOVIEW FV10i). Each positive cell was counted using ImageJ at five wound sites: two sites near the two wound edges and three sites around the center of the wound. The areas of these five sites were measured and calculated using ImageJ. The total number of anti-Ym1-positive (Ym1^+^) M2 macrophages and anti-Mac-3-positive macrophages (Mac-3^+^) at the five sites was divided by the whole area. The ratio of anti-Ym1^+^ in the wound area was calculated using the number of anti-Ym1^+^ M2 macrophages/the number of anti-Mac-3^+^ macrophages in the wound area.

### RNA isolation

RNA was isolated from the whole-wound homogenate using the Purelink RNA mini kit (ThermoFisher Scientific, Waltham, MA, USA). The concentration and purity of RNA were determined using Agilent 2200 Tapestation System (Agilent).

### Microarray profiling

Whole-genome expression profiling was performed using Agilent mouse 4 x 44 K microarrays (Agilent). Briefly, cyanine-3-labeled cRNA was prepared from 0.2 μg RNA using Low Input Quick Amp Labeling Kit (Agilent), according to the manufacturer’s instructions, followed by RNeasy column purification (QIAGEN, N.V., Venlo, Netherlands). Samples were hybridized for 17 h at 65°C in a rotating Agilent hybridization oven. After hybridization, microarrays were washed with GE Wash Buffer 1 and 2 (Agilent) and then dried immediately.

Slides were scanned immediately after washing in the Agilent DNA Microarray Scanner (G2539A) using one color scan setting for 4 x 44k array slides. The scanned images were analyzed with Feature Extraction Software version 11.0.1.1 (Agilent) using default parameters (protocol AgilentHD_GX_1Color and Grid: 026655_D_F_20121126) to obtain a background subtracted and spatially detrended processed signal. Data were normalized and filtered with three filters using GeneSpring version 12.1 (Agilent). The fold change (FC) ratios of normalized data for genes were calculated, and genes with an FC >2.0 were identified. Differentially regulated transcripts were used in Gene Set Enrichment Analysis to identify enriched functional gene oncology annotations with GeneSpring version 12.1.

### Statistical analysis

Data were expressed as mean ± standard error of the mean (SEM) and were analyzed using JMP^®^ 12.1.0 (SAS Institute Inc., Cary, NC, USA). The means among multiple groups were compared via one-way analysis of variance (ANOVA), followed by post hoc pairwise comparisons using the Tukey–Kramer multiple comparison test. For the microarray data, significantly differentially expressed genes (DEGs) were identified using the moderated *t*-test. Volcano plots were used to represent the DEGs between two different conditions with the aim to visualize the relationship between FC and statistical significance. A p- value *<* 0.05 was considered to indicate statistical significance.

## Results

### Blood glucose level before and after fasting before wounding

**Fig 1:**
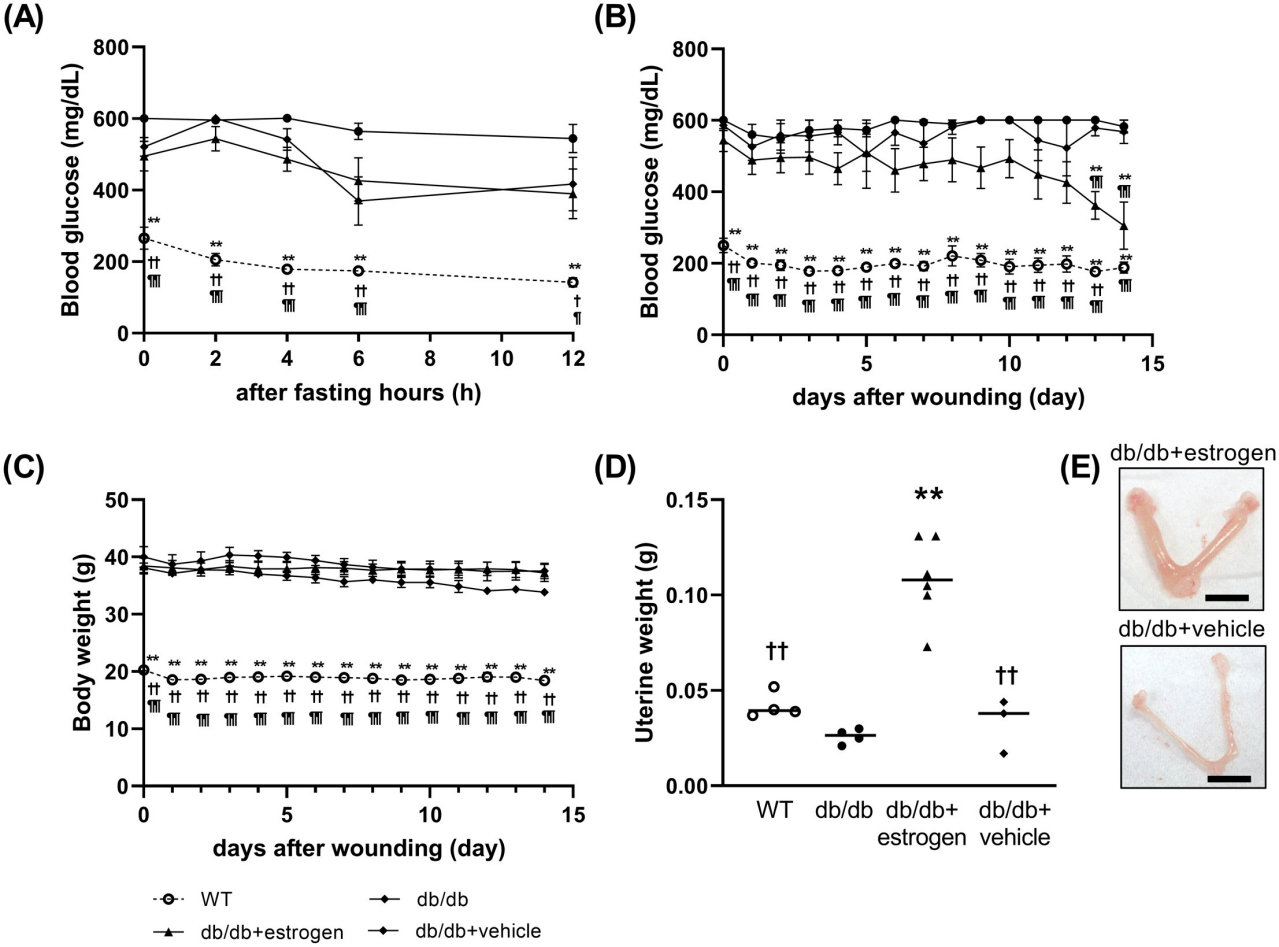
Blood glucose, body weight, and uterine weight. (A) Hourly blood glucose levels between 0 (initial) and 12 h after fasting are presented as line graphs. (B) Daily blood glucose levels after wounding are shown as line graphs. (C) Daily body weights after wounding are depicted as line graphs. (D) Uterine weights on day 14 after wounding are shown as dotted graphs. Values are expressed as means ± SEM, n = 3–6; ANOVA, Tukey’s honestly significant difference (HSD) tests, **p < 0.01: versus the db/db group, ^†^p < 0.05 and ^††^p < 0.01: versus the db/db + estrogen group, and ^¶^p < 0.05, ^¶¶^p < 0.01 versus the db/db + vehicle group. (E) Macroscopic images of the uterus.

Before wounding, the blood glucose levels were monitored before and after fasting (2–12 h), respectively. The blood glucose levels before fasting were 265.8 ± 30.7, 600.5 ± 0.5, 494.7 ± 41.9, and 520.7 ± 26.3 mg/dL in the WT, db/db, db/db + estrogen, and db/db + vehicle groups, respectively. The blood glucose levels before fasting were significantly higher in the db/db, db/db + estrogen, and db/db + vehicle groups than in the WT group (p < 0.01). After fasting, the blood glucose levels gradually decreased in all groups. The blood glucose levels 12 h after fasting were 142.5 ± 12.8, 544.5 ± 39.6, 389.7 ± 69.4, and 417.0 ± 74.7 mg/dL in the WT, db/db, db/db + estrogen, and db/db + vehicle groups, respectively (Fig 1A).

### Blood glucose levels after wounding

On day 0 after wounding, the blood glucose levels were 250.0 ± 20.0, > 601, 544.8 ± 32.3, and 586.3 ± 14.7 mg/dL in the WT, db/db, db/db + estrogen, and db/db + vehicle groups, respectively. The blood glucose levels on day 0 after wounding were significantly higher in the db/db, db/db + estrogen, and db/db + vehicle groups than in the WT group (p < 0.01). The blood glucose levels in the db/db, db/db + vehicle, and WT groups were consistent during the observational period. By contrast, the blood glucose level in the db/db + estrogen group gradually decreased, and it was significantly lower than in the db/db and db/db + vehicle groups on days 13 and 14 (p < 0.01 and p < 0.05, respectively) (Fig 1B).

### Body weight after wounding

On day 0 after wounding, the body weights in the WT, db/db, db/db + estrogen, and db/db + vehicle groups were 20.3 ± 0.5, 40.0 ± 1.8, 38.4 ± 1.4, and 38.1 ± 0.9 g, respectively. Invasion caused by wounding, resulted in a decline in the body weight of all groups. On day 14, the body weights in the WT, db/db, db/db + estrogen, and db/db + vehicle groups were 18.4 ± 0.5, 37.6 ±1.3, 37.2 ± 1.5, and 33.9 ± 0.7 g, respectively. The body weights were significantly higher in the db/db, db/db + estrogen, and db/db + vehicle groups than in the WT group on days 0–14 (p < 0.01) (Fig 1C).

### Uterine weight

The db/db + estrogen group displayed a significantly larger uterine weight than the WT, db/db, and db/db + vehicle groups on day 14 (p < 0.01) (Fig 1D and 1E).

### Wound area

**Fig 2:**
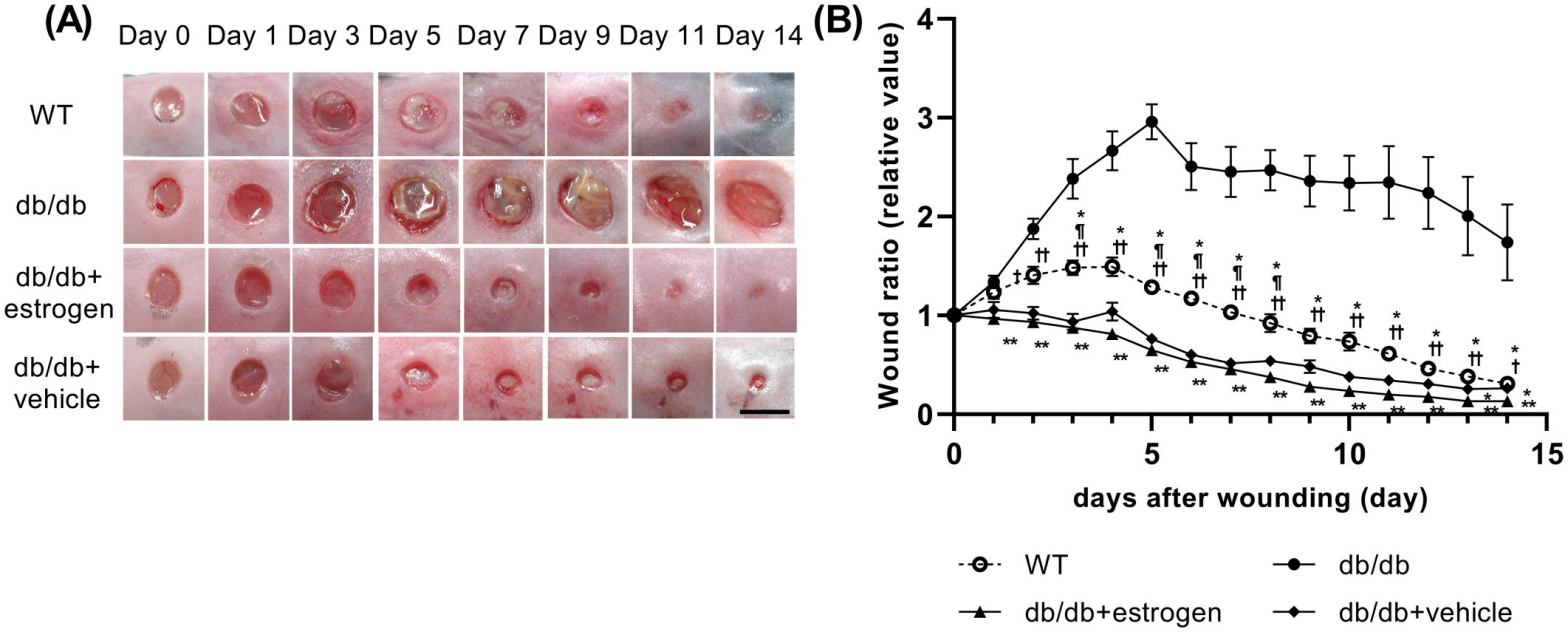
Macroscopic wound healing. (A) Wounds with a diameter of 4 mm were inflicted, and images were obtained to assess healing. Bar, 5 mm. (B) The wound area-to-the initial area ratios on day 0 are shown as line graphs based on each day. Values were expressed as mean ± SEM, n = 6–12; ANOVA, Tukey’s HSD test, *p < 0.05, **p < 0.01: versus the db/db group, ^†^p < 0.05, ^††^p < 0.01: versus the db/db + estrogen group, and ^¶^p < 0.05 versus the db/db + vehicle group.

The wound areas in the WT group increased for 4 days (ratio of the wound area to the initial wound area on day 4: 1.49 ± 0.07) and then rapidly decreased until day 12. Then, they decreased gradually until day 14 (0.31 ± 0.03). The wound areas in the db/db group expanded for 5 days (2.96 ± 0.18) and then somewhat gradually decreased until day 14 (1.74 ± 0.38). By contrast, the wound areas in the db/db + estrogen group did not increase, but rapidly decreased until day 10. Then, they decreased gradually until day 14 (0.13 ± 0.02). In addition, the wound areas in the db/db + vehicle group slightly increased on day 4 (1.04 ± 0.09) and then rapidly decreased until day 10. Then, they decreased gradually until day 14 (0.26 ± 0.05) (Fig 2A and 2B). The wound area ratio was significantly larger in the db/db group than in the WT group on days 3–14 (p < 0.05). By contrast, wound area ratios were significantly lower in the db/db + estrogen and db/db + vehicle groups than in the db/db group on days 1–14 (p < 0.01) and 2–14 (p < 0.05), respectively. Moreover, wound area ratios were significantly lower in the db/db + estrogen and db/db + vehicle groups than in the WT group on days 1–14 (p < 0.05) and 3, 5–8 (p < 0.05), respectively (Fig 2B).

### Re-epithelialization and angiogenesis

**Fig 3:**
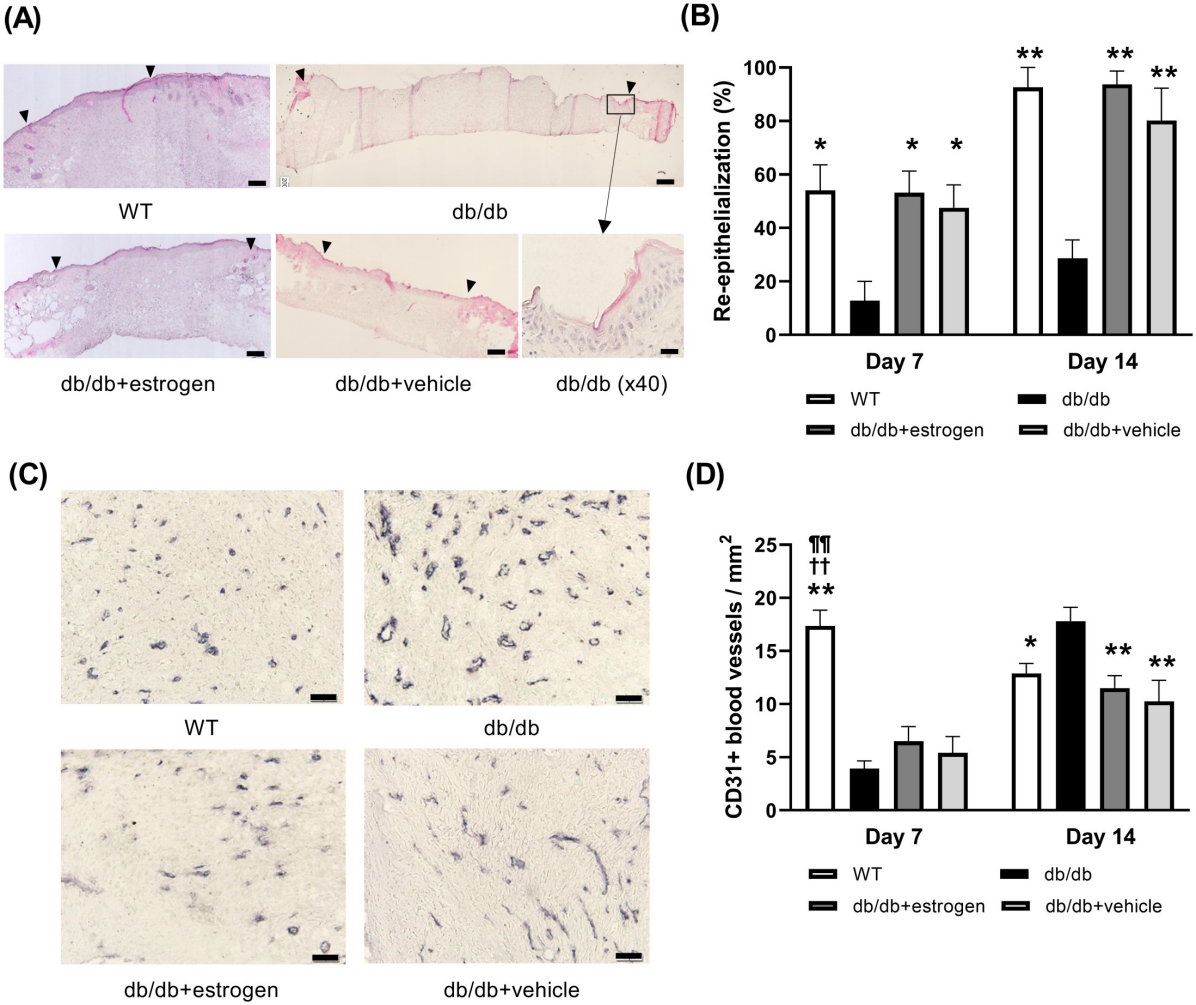
Re-epithelialization and new blood vessels. (A) H&E staining (bars, 200 μm and 20 μm for db/db [40×]) of granulation tissues on day 14 and (B) the ratio of re-epithelialization (%) are depicted as box graphs. (C) New blood vessels stained with an anti-CD31 antibody (bars, 200 μm) were observed in granulation tissues on day 14 and (D) the ratio of new blood vessels (%) are depicted as box graphs. Values are expressed as mean ± SEM, n = 5–10; ANOVA, Tukey’s HSD tests, *p < 0.05, **p < 0.01 versus the db/db group, ^††^p < 0.01: versus the db/db + estrogen group, and ^¶¶^p < 0.01: versus the db/db + vehicle group.

In the WT group, the new epithelium gradually elongated from the wound edges and was nearly completely covered with the wound surface until day 14. By contrast, in the db/db group, the new epithelium was somewhat elongated from the wound edges. However, it was not covered with the wound surface completely on day 14. After treatment with topical estrogen, the new epithelium rapidly elongated from the wound edges, and it was nearly completely covered with the wound surface until day 14 in the db/db + estrogen group. In the db/db + vehicle group, the new epithelium elongated from the wound edges. However, it was not covered with the wound surface completely until day 14. The re-epithelialization ratio was significantly lower in the db/db group than in the WT group on days 7 and 14 (p < 0.05 and p < 0.01, respectively). By contrast, it was significantly higher in the db/db + estrogen group than in the db/db group on days 7 and 14 (p < 0.05 and p < 0.01, respectively). Moreover, the db/db + vehicle group had a significantly higher re-epithelialization ratio than in the db/db group on days 7 and 14 (p < 0.05 and p < 0.01, respectively) (Fig 3A and 3B).

In the WT group, several new blood vessels were synthesized from the wound edges and the bottom of the wound on day 7, and they gradually disappeared until day 14. In the db/db group, the new blood vessels were not synthesized from the wound edges and the bottom of the wound on day 7. However, they were gradually synthesized until day 14. In the db/db + estrogen and db/db + vehicle groups, the new blood vessels were synthesized at a later time compared with the WT group, and they gradually disappeared until day 14. The ratio of new blood vessels was significantly lower on day 7 (p < 0.01) and higher on day 14 (p < 0.05) in the db/db group than in the WT group. By contrast, the ratios of new blood vessels were significantly lower in the db/db + estrogen and db/db + vehicle groups than in the WT group on day 7 (p < 0.01) and in the db/db group on day 14 (p < 0.01) (Fig 3C and 3D).

### Macrophages and Ym1+ M2 macrophages

**Fig 4:**
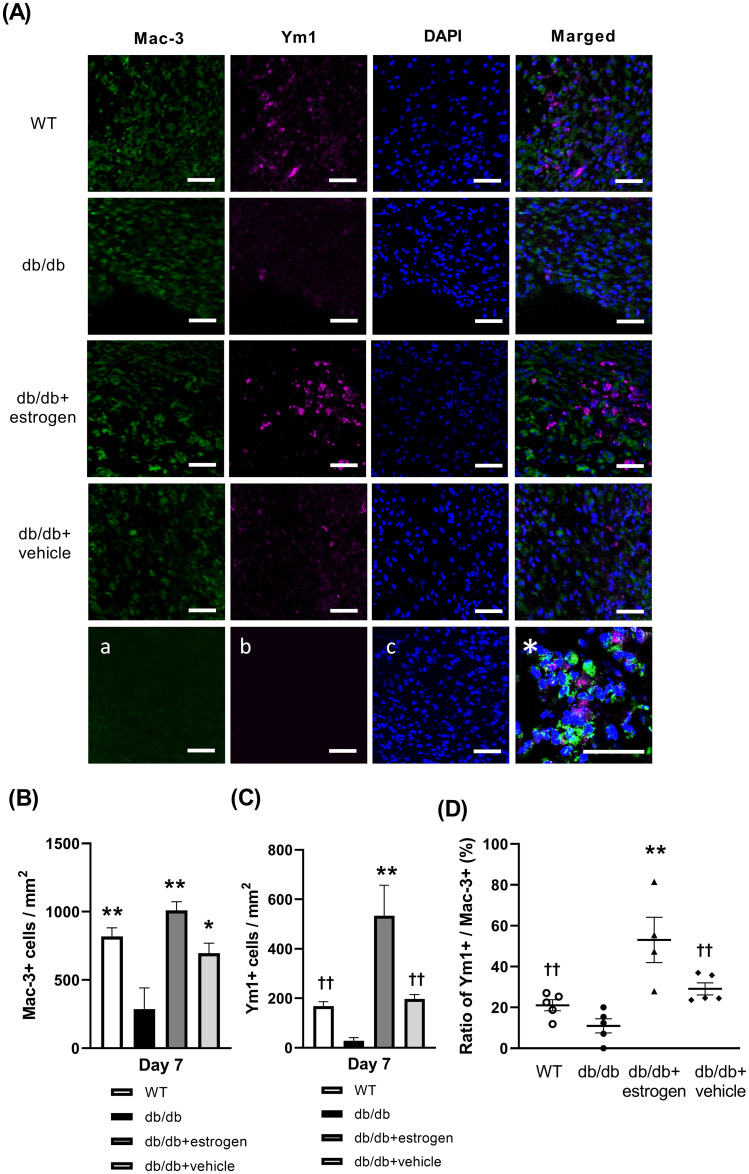
Macrophages and ym1^+^ M2 macrophages. (A) Immunofluorescence staining with Alexa Fluor 488 (green; anti-mac-3 antibody), Alexa Fluor 647 (pink; anti-ym1 antibody), and DAPI (blue) were observed on day 7. The panels labeled as a, b and c are negative controls without primary antibodies, and the panel labeled with an asterisk is an enlarged representative image of the db/db + estrogen group. Bars, 50 μm. (B) The number of macrophages per mm^2^ and (C) the number of ym1^+^ M2 macrophages per mm^2^ are depicted in box graphs. (D) The ratio of ym1^+^ M2 macrophages/macrophages (%) is shown in doted graphs. Values were expressed as mean ± SEM, n = 4–5; ANOVA, Tukey’s HSD test, *p < 0.05, **p < 0.01: versus the db/db group, ^††^p < 0.01: versus the db/db + estrogen group.

The wounds in the WT, db/db + estrogen, and db/db + vehicle groups had a large number of macrophages on day 7. Meanwhile, the wounds in the db/db group had a low number of macrophages on day 7. There was a large number of Ym1^+^ M2 macrophages in the wounds in the WT and db/db + vehicle groups on day 7 and particularly the db/db + estrogen group. By contrast, the wounds in the db/db group had a small number of Ym1^+^ M2 macrophages on day 7. The number of macrophages was significantly lower in the db/db group than in the WT, db/db + estrogen, and db/db + vehicle groups (p < 0.01, p < 0.01, and p < 0.05, respectively) (Fig 4A and 4B). The number of Ym1^+^ M2 macrophages was significantly larger in the db/db + estrogen group than in the db/db group on day 7 (p < 0.01). Furthermore, it was significantly larger in the db/db + estrogen group than in the WT and db/db + vehicle groups on day 7 (p < 0.01) (Fig 4A and 4C). In addition, the Ym1^+^ M2 macrophage/macrophage ratio was significantly higher in the db/db + estrogen group than in the db/db group on day 7 (p < 0.01). Moreover, it was significantly higher in the db/db + estrogen group than in the WT and db/db + vehicle groups on day 7 (p < 0.01) (Fig 4A and 4D).

### Microarray analysis of global gene expression changes

**Fig 5:**
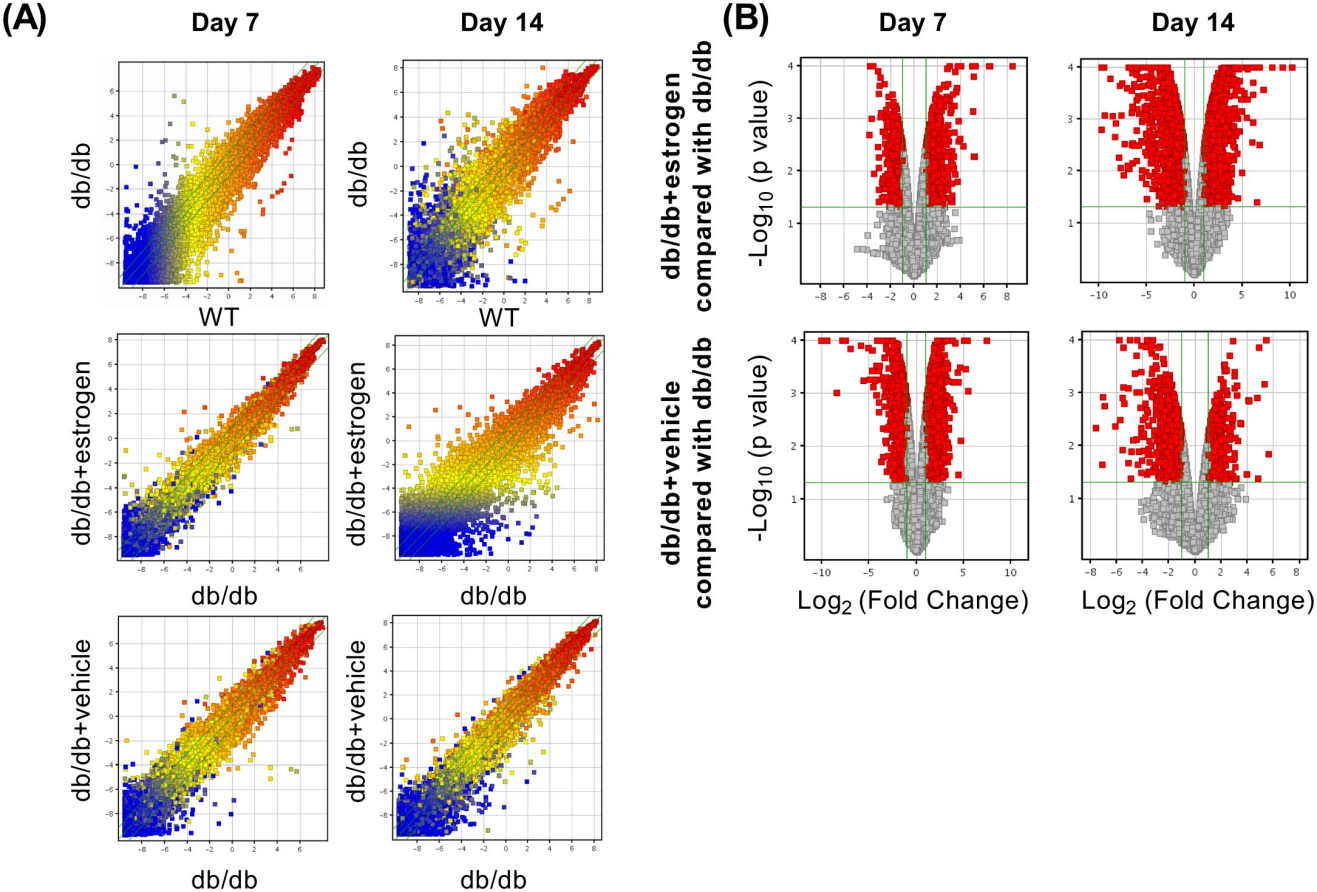
Scatter and volcano plots. (A) The scatter plots on days 7 and 14 after wounding are shown. The upregulated and downregulated genes are depicted in red and blue, respectively. (B) The volcano plots show DEG with a p- value <0.05 and an FC >2.0 on days 7 and 14 after wounding, depicted in red, in the db/db + estrogen or the db/db + vehicle group compared with the db/db group.

Compared with the WT group, the db/db group had a large gene expression variation both on days 7 and 14. Meanwhile, the global gene expression in the db/db + estrogen group compared with the db/db group had small and moderate gene expression variations on days 7 and 14, respectively. The global gene expression in the db/db + vehicle group compared with the db/db group had moderate and small gene expression variations on days 7 and 14, respectively (Fig 5A). Tables 1 and 2 depict the top 10 upregulated or downregulated genes, respectively. These genes were somewhat reversed by estrogen treatment, especially on day 14, in the db/db group compared with the WT group.

**Table 1:** Top 10 upregulated genes in db/db mice.

Number	Symbol	Description	db/db^a^ (FC)	db/db+ estrogen^b^ (FC)	db/db+ vehicle^b^ (FC)
Day 7					
XM_003689292	*LOC100862557*	myosin-6-like	1703.89	-	−1123.04
NM_080728	*Myh7*	myosin, heavy polypeptide 7	677.30	-	−706.18
NM_010861	*Myl2*	myosin, light polypeptide 2	455.36	-	−345.34
NM_022314	*Tpm3*	tropomyosin 3	365.62	-	−236.57
NM_008571	*Mcpt2*	mast cell protease 2	268.07	−2.05	-
NM_018803	*Syt10*	synaptotagmin X	243.66	-	-
NM_010871	*Naip6*	apoptosis inhibitory protein 6	218.05	-	-
NM_010859	*Myl3*	myosin, light polypeptide 3	109.86	-	−186.16
NM_001085378	*Myh7b*	myosin, heavy chain 7B	102.49	-	−127.80
NM_010381	*H2-Ea-ps*	histocompatibility 2, class II antigen E alpha	61.42	-	-
Day 14					
NM_181596	*Retnlg*	resistin like gamma	818.32	−314.14	−8.09
NM_018803	*Syt10*	synaptotagmin X	813.82	-	-
NM_008694	*Ngp*	neutrophilic granule protein	685.53	−823.08	−131.23
NM_008571	*Mcpt2*	mast cell protease 2	469.72	-	-
NM_008522	*Ltf*	lactotransferrin	281.25	−452.90	−120.05
NM_011260	*Reg3g*	regenerating islet-derived 3 gamma	197.44	−22.86	−5.46
AY500847	*Mpo*	myeloperoxidase	147.05	−78.07	−21.04
NM_009921	*Camp*	cathelicidin antimicrobial peptide	135.06	−135.13	−67.88
XM_003689292	*LOC100862557*	myosin-6-like	106.89	−375.24	-
NM_207534	*Mrgpra5*	MAS-related GPR	92.83	−87.92	−8.18

Data were expressed in p- values <0.05 and FCs >2.0 compared with a: the WT group and b: the db/db group. FC: Fold change.

**Table 2:** Top 10 downregulated genes in db/db mice.

Number	Symbol	Description	db/db^a^ (FC)	db/db+ estrogen^b^ (FC)	db/db+ vehicle^b^ (FC)
Day 7					
NM_177578	*Skint3*	selection and upkeep of intraepithelial T cells 3	−1493.09	-	-
NM_145585	*Thumpd1*	THUMP domain containing 1	−1421.40	-	-
NM_178786	*Skint4*	selection and upkeep of intraepithelial T cells 4	−296.08	-	3.21
NM_001012323	*Mup20*	major urinary protein 20	−136.16	2.03	-
NM_001126319	*Mup9*	major urinary protein 9	−122.09	-	4.70
NM_020000	*Med8*	mediator of RNA polymerase II transcription	−118.45	-	-
NM_008649	*Mup5*	major urinary protein 5	−111.84	-	-
NM_001200006	*Mup17*	major urinary protein 17	−109.79	-	-
NM_001039544	*Mup3*	major urinary protein 3	−105.14	-	-
NM_001135127	*Mup19*	major urinary protein 19	−98.09	-	-
Day 14					
NM_177578	*Skint3*	selection and upkeep of intraepithelial T cells 3	−2367.63	-	2.68
NM_145585	*Thumpd1*	THUMP domain containing 1	−1240.81	-	-
NM_178786	*Skint4*	selection and upkeep of intraepithelial T cells 4	−784.27	7.98	-
MN_133730	*Krt25*	keratin 25	−687.54	1187.09	49.86
NM_001012323	*Mup20*	major urinary protein 20	−328.19	18.63	-
NM_001200006	*Mup17*	major urinary protein 17	−326.94	16.18	-
NM_008649	*Mup5*	major urinary protein 5	−295.54	16.51	-
NM_001199333	*LOC100048884*	novel member of the major urinary protein gene family	−295.04	16.14	-
NM_001126319	*Mup9*	major urinary protein 9	−280.05	5.18	-
NM_001135127	*Mup19*	major urinary protein 19	−273.09	14.84	-

Data were expressed in p- values <0.05 and FCs >2.0 compared with a: the WT group and b: the db/db group. FC: Fold change.

### Microarray analysis of the global gene expression of estrogen administration-specific changes

**Fig 6:**
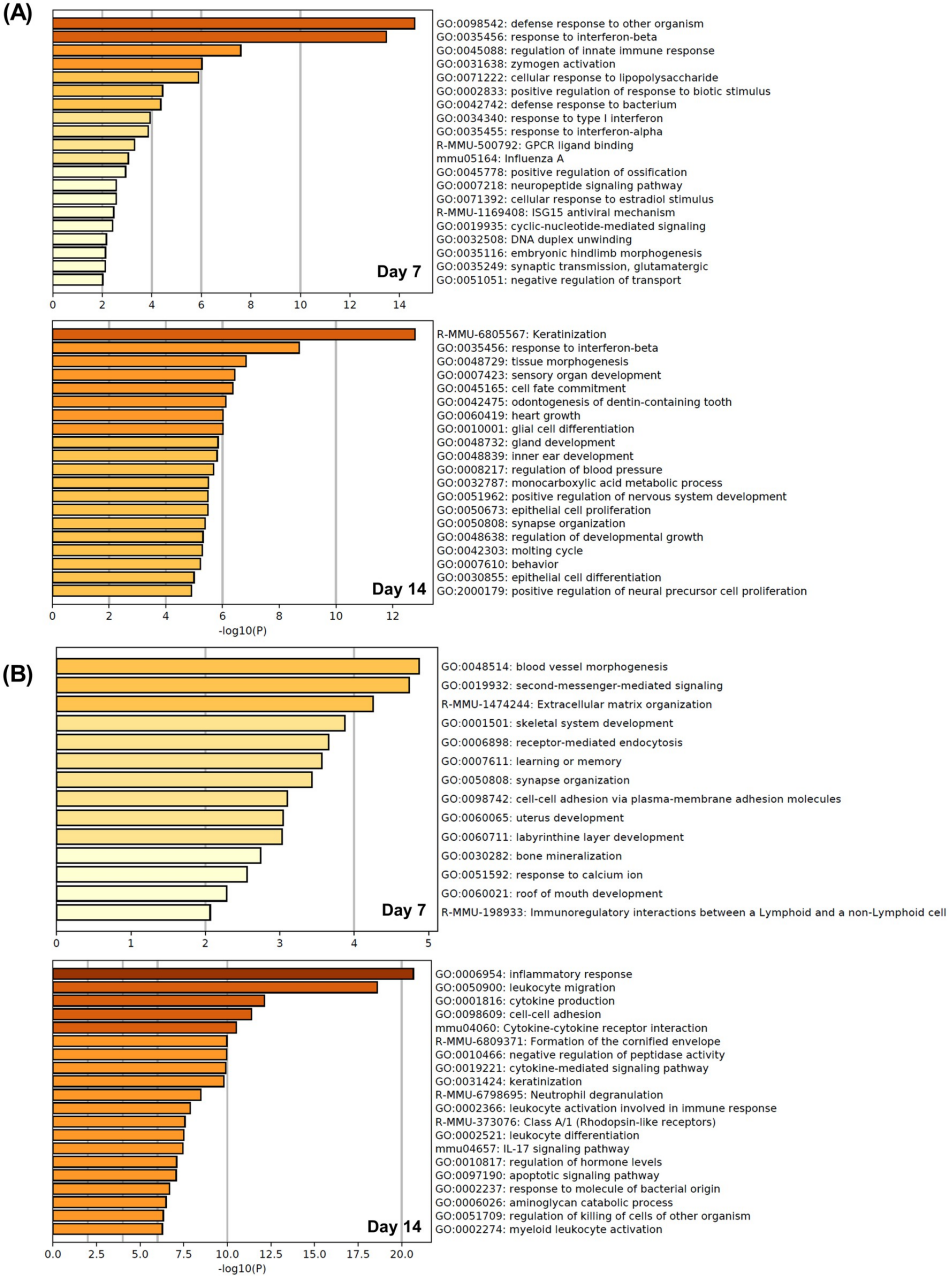
Gene set enrichment analysis. (A) Gene set enrichment analysis of db/db + estrogen-specific upregulation on days 7 and 14 after wounding. (B) Gene set enrichment analysis of db/db + estrogen-specific downregulation on days 7 and 14 after wounding.

The volcano plots were used to visualize the DEG with a p- value <0.05 and an FC >2.0 in the db/db + estrogen or db/db + vehicle group compared with the db/db group on days 7 and 14 (Fig 5B). Tables 3 and 4 show the top 10 upregulated or downregulated genes in db/db + estrogen-specific changes, respectively. Enrichment analysis of db/db + estrogen-specific upregulation revealed overrepresented biological process terms associated with the defense response to other organisms, response to interferon-beta and regulation of innate immune response on day 7, and keratinization, response to interferon-beta and tissue morphogenesis on day 14 (Fig 6A). Moreover, enrichment analysis of db/db + estrogen-specific downregulation revealed overrepresented biological process terms associated with blood vessel morphogenesis, second messenger-mediated signaling, and extracellular matrix organization on day 7 and inflammatory response, leukocyte migration, and cytokine production on day 14 (Fig 6B).

**Table 3:** Top 10 genes upregulated specifically in db/db + estrogen mice.

Number	Symbol	Description	db/db + estrogen specific (FC)
Day 7			
NM_001011849	*Olfr792*	olfactory receptor 792	12.36
NM_010374	*Gzmf*	granzyme F	11.23
NM_146402	*Olfr1303*	olfactory receptor 1303	11.04
NM_198414	*Paqr9*	progestin and adipoQ receptor family member IX	9.65
NM_010640	*Klk1b11*	kallikrein 1-related peptidase b11	9.32
NM_027306	*Zdhhc25*	zinc finger, DHHC domain containing 25	8.53
NM_020268	*Klk1b27*	kallikrein 1-related peptidase b27	7.33
NM_172861	*Slc7a14*	solute carrier family 7	6.28
NM _147079	*Olfr547*	olfactory receptor 547	5.49
NM _080644	*Cacng5*	calcium channel, voltage-dependent, gamma subunit 5	5.42
Day 14			
NM_001037800	*Cd209b*	CD209b antigen	113.77
NM_010668	*Krt2*	keratin 2	91.67
NM_027983	*Krt33a*	keratin 33A	66.70
NM_020509	*Retnla*	resistin like alpha	44.74
NM_026956	*Cd209f*	CD209f antigen	42.02
NM_033217	*Ngfr*	nerve growth factor receptor	36.75
NM_027574	*Krt28*	keratin 28	32.90
NM_024450	*Scd3*	stearoyl-coenzyme A desaturase 3	32.25
NM_027574	*Krt28*	keratin 28	31.68
NM_175448	*Clvs2*	clavesin 2	31.24

Data were expressed in p-values <0.05 and displayed by excluding predicted genes or RIKEN cDNA genes. FC: fold change.

**Table 4:** Top 10 genes downregulated specifically in db/db + estrogen mice.

Number	Symbol	Description	db/db + estrogen specific (FC)
Day 7			
NM_ 001003667	*Krt77*	keratin 77	−8.26
NM_ 008973	*Ptn*	pleiotrophin	−5.47
NM_153539	*Fam5c*	family with sequence similarity 5, member C	−5.26
BC115767	*Adam19*	a disintegrin and metallopeptidase domain 19	−4.49
NM_ 009575	*Zic3*	zinc finger protein of the cerebellum 3	−3.98
NM_ 008263	*Hoxa10*	homeobox A10	−3.81
NM_ 001109758	*Bcan*	brevican	−3.81
NM_ 144551	*Trib2*	tribbles homolog 2	−3.63
NM _ 001162476	*Stra6*	stimulated by retinoic acid gene 6	−3.62
NM _001033354	*Iqsec3*	IQ motif and Sec7 domain 3	−3.54
Day 14			
NM _011472	*Sprr2f*	small proline-rich protein 2F	−817.58
NM_ 011475	*Sprr2i*	small proline-rich protein 2I	−699.84
NM_001039594	*Lce3a*	late cornified envelope 3A	−673.86
XM_003689292	*LOC100862557*	myosin-6-like	−375.24
NR_003185	*Sprr2j-ps*	small proline-rich protein 2J, pseudogene	−294.32
NM_011477	*Sprr2k*	small proline-rich protein 2K	−235.77
NR_003548	*Sprr2g*	small proline-rich protein 2G	−217.35
NM_ 203320	*Cxcl3*	chemokine (C-X-C motif) ligand 3	−213.67
NM_ 080728	*Myh7*	myosin, heavy polypeptide 7	−202.12
NM_ 019471	*Mmp10*	matrix metallopeptidase 10	−157.10

Data were expressed in p-values <0.05 and displayed by excluding predicted genes or RIKEN cDNA genes. FC: fold change.

## Discussion

Chronic nonhealing wounds are a major health and economic burden worldwide. Recently, female sex hormones have been found to be beneficial on wound healing in diabetes [17]. However, whether estrogen treatment can promote cutaneous wound healing in diabetes remains unclear. Therefore, the present study aimed to assess the effect of estrogen on cutaneous wound healing in a type 2 diabetes db/db mice model. Results showed that estrogen treatment promoted cutaneous wound healing in db/db mice.

The levels of ovarian steroid hormones, which include serum estradiol and progesterone, were consistently lower in db/db mice than in their littermate controls [28]. Zhuge et al. showed that the uterine weight of estrogen-treated db/db mice was significantly higher than that of placebo-treated db/db mice [18]. In the current study, the uterine weight in the db/db + estrogen group was significantly higher than those in the db/db, db/db + vehicle, and WT groups. Estradiol was correlated with uterine weight; estrogen induced increase in uterine weight [29, 30], and successful estrogen replacement was confirmed by a higher uterine weight [16, 27]. Therefore, estrogen replacement was effective in the current study.

Previous studies have shown that mice with blood glucose levels of > 250 mg/dL [31, 32], 300 mg/dL [33, 34], or 350 mg/dL [35] were considered to have chemically streptozotocin-induced type 1 diabetes. Moreover, in db/db mice with genetically induced type 2 diabetes, the blood glucose level was over 400 mg/dL after the 8^th^ week [28, 36]. In the current study, the blood glucose levels were > 601 mg/dL in the db/db group, and they remained constant during the observational period. Therefore, our model was confirmed to have diabetes. In contrast, in the current study, the blood glucose level in the db/db + estrogen group was significantly lower than those in the db/db and db/db + vehicle groups. Previous studies have shown that estrogen treatment normalized metabolic homeostasis while maintaining systemic euglycemia in db/db mice [37, 38]. Garris revealed that the severity of genomic db/db mutation expression may be modified by estrogen, with the gonadal steroid probably acting as a nuclear-specific stimulatory transcriptional modulator of cellular glucometabolic cascades [39]. Therefore, in the current study, topical estrogen application could regulate hyperglycemia.

Diabetes mellitus impairs cutaneous wound healing, thereby making patients with type 2 diabetes susceptible to chronic nonhealing DFU that often result in limb amputations [7, 8, 40]. In animal models, wound healing was impaired in db/db mice with genetically induced type-2 diabetes [41, 42]. The current study showed that the wound ratio in the db/db group was significantly larger than that in the WT group. A histological analysis revealed a longer distance between the new epithelium tips on days 7 and 14 and a low density of new blood vessels on day 7 compared with the WT group. This result indicates that re-epithelialization and angiogenesis showed significant impairment in mice with type 2 diabetes. Therefore, cutaneous wound healing was delayed in mice with type 2 diabetes. By contrast, this delay was reversed by estrogen application in db/db mice. The wound ratios in the db/db + estrogen group were significantly lower than that in the db/db group. A histological analysis in the db/db + estrogen group revealed a longer distance between the new epithelium tips on days 7 and 14, and an early withdrawal of new blood vessels on day 14 compared with the db/db group. This finding indicated that re-epithelialization and angiogenesis were significantly promoted by estrogen application in mice with type 2 diabetes. Previous studies reported that estrogen treatment accelerated wound healing by reducing wound area and improving neovascularization in db/db mice with type 2 diabetes [18], and the administration of estrogen improved vascular outcomes in rats with STZ-induced type 1 diabetes [43]. Therefore, the present study showed that delayed cutaneous wound healing improved after estrogen treatment in mice with type 2 diabetes.

Macrophage infiltration was delayed in diabetic wounds [44]. In the current study, the db/db group had a significantly smaller number of macrophages than the WT group. This result showed that macrophage infiltration was delayed in db/db mice. Moreover, the function and morphology of macrophages are also disturbed in db/db mice [45]. Macrophages can have different functional phenotypes, which are divided into two groups: pro-inflammatory M1 macrophages and anti-inflammatory M2 macrophages. In diabetic wounds, M1 macrophages include most macrophages in the wounds of db/db mice with type 2 diabetes, which reflected the improvement of M1 macrophages and the suppression of M2 macrophages [41], and dominant phenotype impairment in tissue repair [46, 47]. In the present study, the Ym1^+^ M2 macrophage/macrophage ratio was significantly larger in the db/db + estrogen group than in the db/db group on day 7. Some previous studies showed that estrogen application promotes M2 macrophage appearance [27, 48, 49]. M2 macrophages can promote the migration and proliferation of keratinocytes, fibroblasts, and endothelial cells [50, 51]. Moreover, experimental therapies used at present have examined the role of macrophages in promoting wound healing in chronic wounds [52]. Therefore, taken together, delayed cutaneous wound healing was improved by topical estrogen application in mice with type 2 diabetes by increasing the number of M2 macrophages in wounds. Thus, further studies should be conducted for identifying the key regulator that can ultimately result in progress in wound healing therapies in chronic nonhealing wounds.

The microarray technology can facilitate the simple and precise detection of substantial changes in gene expression [53]. By profiling this analysis, our dataset provides better insights about key biological processes that occur during wound healing by estrogen treatment. In the current study, the top 10 upregulated or downregulated genes in the db/db group were somewhat reversed by estrogen treatment, particularly on day 14. In addition, another analysis revealed the top 10 upregulated or downregulated genes in db/db + estrogen-specific changes on days 7 and 14. Several genes previously mentioned were confirmed as key regulators in cutaneous wound healing. Cd209 antigens and resistin-like alpha (*Retnla*), which is also found in inflammatory zone 1 (*Fizz 1*), are considered M2 macrophage markers [54]. Tissue kallikreins are key genes that promote re-epithelialization [55–57]. Recently, the use of tissue kallikreins and its receptors as therapeutic targets for chronic nonhealing wounds in diabetes attracted attention [56–59]. Furthermore, the enrichment analysis showed key regulated biological processes promoted by estrogen treatment in db/db mice. These results may explain that the differential regulation of these genes via estrogen treatment improves wound healing in db/db mice. Nevertheless, further studies should be conducted to evaluate the effect of these genes on cutaneous wound healing and identify critical genes involved in the regulation of these events.

In summary, topical estrogen application reduced the wound area, promoted re-epithelialization and angiogenesis, and increased the number of M2 macrophages in type 2 diabetes. Furthermore, this treatment improved the differential regulation of genes in db/db mice. Therefore, it could promote cutaneous wound healing in female mice with type 2 diabetes. We believe that our results will be beneficial for wound care among women with diabetes. This is the first study specifically elucidating the effect of topical estrogen application on cutaneous wound healing in the db/db mouse model of type 2 diabetes. The effect of estrogen analogs must be further investigated in this model to better understand the role of estrogens in cutaneous wound healing.
